# Evaluation of the Vmaxpro sensor for assessing movement velocity and load-velocity variables: accuracy and implications for practical use

**DOI:** 10.5114/biolsport.2024.125596

**Published:** 2023-05-25

**Authors:** Boris Dragutinovic, Mats W. Jacobs, Joshua F. Feuerbacher, Jan-Peter Goldmann, Sulin Cheng, Moritz Schumann

**Affiliations:** 1 Department of Molecular and Cellular Sports Medicine, Institute of Cardiovascular Research and Sports Medicine, German Sport University, Cologne, Germany; 2 Institute of Biomechanics and Orthopaedics, German Sport University Cologne, Cologne, Germany; 3 German Research Centre of Elite Sport Cologne, Cologne, Germany; 4 Department of Physical Education, Exercise, Health and Technology Centre, Shanghai Jiao Tong University, Shanghai, China; 5 Exercise Translational Medicine Centre, Shanghai Centre for Systems Biomedicine, Shanghai Jiao Tong University, Shanghai, China; 6 Division of Training and Movement Science, University of Potsdam, Potsdam, Germany; 7 Faculty of Sport and Health Sciences, University of Jyväskylä, Jyväskylä, Finland

**Keywords:** Validity, Reliability, Velocity-based training, Inertial measurement unit, Strength training

## Abstract

We investigated the ecological validity of an inertial measurement unit (IMU) (Vmaxpro) to assess the movement velocity (MV) during a 1-repetition maximum (1RM) test and for the prediction of load-velocity (*L-V*) variables, as well as the ecological intra- day and inter-day reliability during free-weight bench press (BP) and squat (SQ). Furthermore, we provide recommendations for the practical use of the sensor. Twenty-three strength-trained men completed an incremental 1RM test, whereas seventeen men further participated in another 3 sessions consisting of 3 repetitions with 4 different loads (30, 50, 70 and 90% of 1RM) to assess validity and intra- and inter-day reliability, respectively. The MV was assessed using the Vmaxpro and a 3D motion capture system (MoCap). *L-V* variables and the 1RM were calculated based on submaximal velocities. The Vmaxpro showed high validity during the 1RM test for BP (*r* = 0.935) and SQ (*r* = 0.900), but with decreasing validity at lower MVs. The *L-V* variables and the 1RM demonstrated high validity for BP (*r* = 0.808–0.942) and SQ (*r* = 0.615–0.741) with a systematic overestimation. Coefficients of variance for intra- and inter-day reliability ranged from 2.4% to 9.7% and from 3.2% to 8.6% for BP and SQ, respectively. The Vmaxpro appears valid at high and moderately valid at low MVs. Depending on the required degree of accuracy, the sensor may be sufficient for the prediction of *L-V* variables and the 1RM. Our data indicate the sensor to be suitable for monitoring changes in MVs within and between training sessions.

## INTRODUCTION

Measuring movement velocity during strength training has become an increasingly important and versatile method for training monitoring and evaluation in both athletic [1] and clinical [2] populations. For example, training loads may be individualised by relative veloc-ity loss thresholds [1], leading to favourable power adaptations com-pared to traditional strength training based on the percentage of the 1-repetition maximum (1RM) [3]. Furthermore, due to the linear load-velocity (*L-V*) relationship in multi-joint exercises, the determi-nation of velocity at submaximal loads (i.e. within 30–80% of the 1RM) can be used as a time-efficient non-demanding method to predict the 1RM [4]. In line with this, other *L-V* relationship variables can provide a more complete evaluation of neuromuscular capacities. As such, the load-axis intercept (i.e., load at zero velocity: *L*_0_), the velocity-axis intercept (i.e., velocity at zero load: *v*_0_) and the area under the line of the *L-V* relationship (*A*_line_ = *L*_0_ · *v*_0_/2) can be used to evaluate the ability of muscles to produce maximal force, velocity and power, respectively [5].

Optical 3-dimensional (3D) motion capture systems (MoCap, e.g. Vicon) are considered the gold standard for assessing movement velocity [6]. However, since this method is labour intensive and expensive, more practical technologies such as linear position-velocity transducers (e.g. GymAware, T-Force) and wearable wireless inertial measurement units (IMUs, e.g. Myotest, OUTPUT) are gaining popularity. Apart from the portability as well as the simplicity of use, linear position-velocity transducers and IMUs have the advantage of providing direct feedback on the movement velocity. In turn, using feedback was shown to have a positive impact on the movement velocity [7], possibly resulting in better power adaptations [8]. While linear position-velocity transducers are attached to the body or the barbell by a cable extension, IMUs are wireless and more cost-effective. However, IMUs are based on the combination of signals from multiple sensors (i.e., accelerometers, gyroscope and magnetic sensors) to estimate movement velocity, and therefore the validity and reliability of IMUs should be considered carefully [9].

One commercially available triaxial IMU that is gaining popularity among practitioners worldwide is the Vmaxpro sensor (Blaumann & Meyer-Sports Technology UG, Magdeburg, Germany). However, scientific data on the validity and the reliability of the Vmaxpro are lacking. In our previous study, the sensor showed high validity (*R*^2^ = 0.935) compared to the criterion device (i.e., Vicon) and moderate to high interclass correlation coefficients (ICCs) for intra-day (ICC: 0.662–0.938; *p* ≤ 0.05) and inter-day reliability (ICC: 0.568–0.837; *p* ≤ 0.05) for the evaluation of mean velocity (MV) in the deep squat (SQ) [10]. The data, however, were obtained in a Smith machine with a guided barbell pathway. One advantage of IMUs is their capability to assess movement velocity in three axes, and the validity and the reliability of IMUs are influenced by the type of the resistance exercise [11].

Considering this, we aimed to investigate the ecological validity and test-retest reliability of the Vmaxpro using free weights in both bench press (BP) and SQ exercise. Furthermore, to better understand how the possible deviation of the Vmaxpro influences training practice, we assessed the validity of the Vmaxpro to predict *L-V* variables (i.e., *L*_0_, *v*_0_, *A*_line_) and the 1RM. Taken together, the aims of our study were to investigate: (1) the ecological validity of the Vmaxpro during an 1RM test; (2) the ecological validity for the prediction of *L-V* variables; (3) the ecological intra-day reliability; and (4) the ecological inter-day reliability.

## MATERIALS AND METHODS

### Study design

**FIG. 1:**
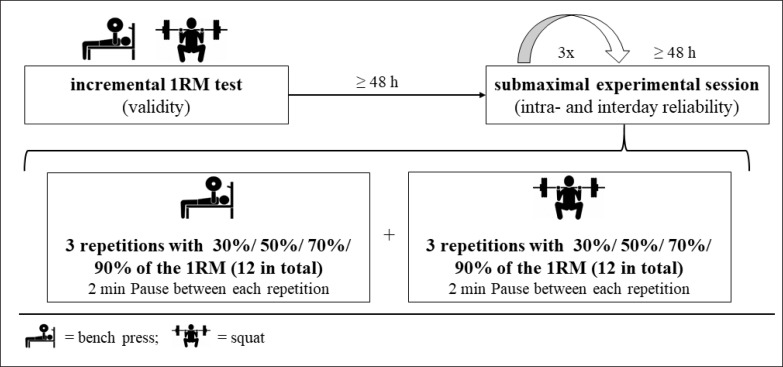
Study design. 1RM = one-repetition maximum.

The study employed a repeated-measures design (Figure 1), with participants completing a total of 4 sessions. During the first visit, participants were familiarised with the BP and SQ protocols and performed an incremental BP and SQ 1RM test. In the following three sessions (i.e. sessions 2–4), the participants performed an experimental session with 3 repetitions at 4 different loads (i.e., 30, 50, 70, and 90% of 1RM). The participants completed all sessions separated by a minimum of 48 h. Throughout all sessions, the MV for BP and SQ was assessed using the Vmaxpro (index device) and a MoCap (Vicon 3D Motion Systems, Oxford, United Kingdom), which was considered as the criterion device.

### Participants

Twenty-three strength-trained men (age: 25 ± 3 years; height: 184.2 ± 7.7 cm; body mass: 82.3 ± 8.2 kg; relative BP 1RM: 1.08±0.21 kg·kg bodyweight^−1^; relative SQ 1RM: 1.37±0.28 kg·kg bodyweight^−1^) completed the incremental 1RM test to determine the ecological validity of the Vmaxpro. Out of these participants, seventeen men (age: 25 ± 3 years; height: 182.6 ± 6.2 cm; body mass: 82.1 ± 5.1 kg; relative BP 1RM: 1.15 ± 0.18 kg · kg bodyweight^−1^; relative SQ 1RM: 1.44 ± 0.26 kg · kg bodyweight^−1^) further participated in the second part of the study, consisting of 3 additional experimental sessions with submaximal loads to determine the intra- and inter-day reliability of the sensor. Participants were eligible to participate in the study if they (1) were non-smokers; (2) had no chronic or acute injuries; (3) were between the ages of 18 and 40; (4) were physically active; and (5) had at least 1 year of strength training experience prior to enrolment in the study. All participants were instructed about possible risks associated with the study. A medical history questionnaire was reviewed and written informed consent was provided prior to inclusion. This study was approved by a local institutional ethic review board and was conducted according to the Declaration of Helsinki.

### Procedures

In order to standardise the execution of the movement throughout every repetition in the 1RM test and the experimental sessions, participants’ standing position and grip width were marked using a tape and the participants’ BP and SQ depth was controlled using safety bars. To reduce the biological within-subject variance, all repetitions were performed with a controlled eccentric phase until the reversal point (i.e., contact of the bar and the safety bar) [12]. Additionally, participants were instructed to hold the position for a momentary pause of 1.5 seconds before performing the concentric phase with maximal velocity [13]. The 1RM was assessed in both BP and SQ (in that order). Testing started with a 5-minute warm-up at an individualised load (i.e., 1.5 times body weight) on a stationary cycle ergometer, followed by 10 repetitions with the unloaded bar (i.e., 20 kg) in both exercises. After performing the initial individualised load (i.e., 30% of the estimated 1RM), the load was progressively increased until the participants were unable to lift the load with the correct technique and without assistance. Every load was performed only once with 2 minutes rest in between. In order to investigate the ecological intra- and inter-day reliability of the Vmax-pro across the entire MV range, the experimental sessions consisted of 3 repetitions at 30, 50, 70, and 90% of the 1RM (12 repetitions in total) for both BP and SQ (in that order), separated by 2 minutes of rest between each repetition. The warm-up and execution of the movement were similar to those performed during the 1RM test.

**FIG. 2:**
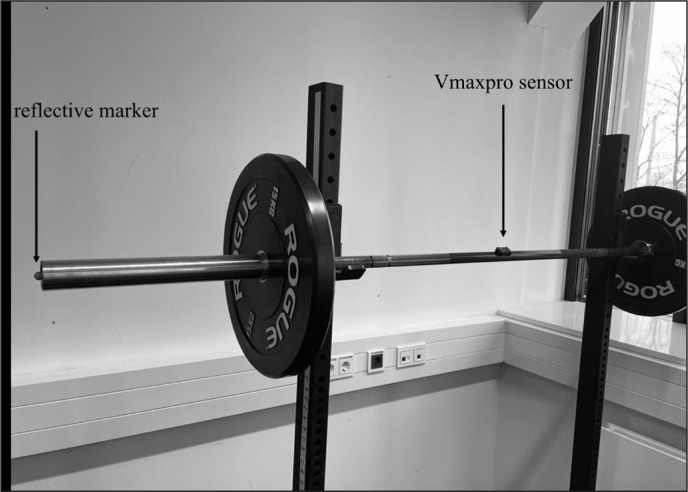
Position of the reflective marker and the Vmaxpro on the barbell.

To obtain the MV of each repetition, the Vmaxpro sensor was attached to the barbell on the basis of the manufacturer’s specifications (i.e. in the centre of the barbell, Figure 2). Instant velocities were recorded at a sampling rate of 200 Hz and were calculated using the Vmaxpro application (version 1.1.4) that was connected to an IOS device (IPhone 11/IPhone 12; Apple, Inc., Cupertino, CA) via a Bluetooth 5.0 connection. Additionally, the MV was obtained using a MoCap with 3 infrared high-speed cameras (Vicon Motion Systems, Oxford, United Kingdom) with 1 reflective marker placed on the end of the barbell (Figure 2). The data were recorded at a frequency of 200 Hz using the software Vicon Nexus (version 2.6). The mean concentric resultant velocities (*v_resultant_*) were then manually calculated by summing up the velocities for all three axes ($v_{resultant}=\sqrt{v_{x}^{2}+v_{y}^{2}+v_{z}^{2}}$). The initiation of the concentric phase was determined at the point where *v_resultant_* > 0, while the end of the concentric phase was defined as the point where *v_resultant_* ≤ 0.

### Calculation of L-V variables and 1RM

Mathematical calculation of the *L-V* variables and the 1RM requires the respective maximal MV at three or more incremental sub-maximal loads, which can be chosen within a range of 30–80% of the estimated 1RM [14]. Therefore, the *L-V* variables were calculated based on the highest MV at 30, 50 and 70% 1RM of each submaximal experimental session (sessions 2–4), for both Vmax and MoCap data. The calculation of the *L-V* variables and the 1RM was based on a least-square linear regression model (*L* [V] = *L*_0_–*sV*). *L*_0_ represents the load at zero velocity and *s* is the slope of the *L-V* relationship. The maximal velocity at zero load (*v*_0_) was calculated as follows: *v*_0_ = *L*_0/*s*_, while the maximal power capacity (*A*_line_) was defined as the area under the *L-V* curve: *A*_line_ = *L*_0_ · *v*_0_/2. For calculation of the predicted 1RM, the following equation was used: 1RM = (*v*_1RM_-*v*_0_)/*s*, where *v_1RM_* is defined as the MV at the maximal load obtained by the MoCap during the incremental 1RM test.

### Statistical analyses

Normality of distribution was assessed by the Shapiro–Wilk test. To enable a differentiated perspective on the validity and reliability of the Vmaxpro as well as to ensure comparability to other studies, multiple measures of validity and reliability were used. For validity analysis, the agreement of the differences between the index and criterion (index – criterion) was assessed by Bland-Altman analysis [15]. Additionally, the Pearson product-moment correlation coefficient *r* was calculated between the index and the criterion for data of the incremental 1RM test, as well as the *L-V* variables (i.e., *L*_0_, *v*_0_, *A*_line_) and the predicted 1RM, and classified as trivial (*r* < 0. 1), low (0.1 ≤ *r* < 0.3), moderate (0.3 ≤ *r* < 0.5), high (0.5 ≤ *r* < 0.7), very high (0.7 ≤ *r* < 0.9), and nearly perfect (*r* ≥ 0.9) [16]. To analyse whether the validity is velocity dependent, additionally a linear regression analysis was performed. Analysis of heteroscedasticity of errors within the linear model was performed using the studentized Breusch-Pagan test. In the case of non-normally distributed residuals, a modified studentized Breusch-Pagan test was performed. Additionally, for validity analysis, the mean absolute percentage error (MAPE) between the index and the criteria was calculated as follows: MAPE = (|Vmax-MoCap|) / Vmax · 100.

In order to provide information on how the possible deviation of the Vmaxpro influences training practice, the proportion of Vmax data of the incremental 1RM test, the submaximal strength sessions (for all loads together and separated by the intensities) and the *L-V* variables (i.e., *L*_0_, *v*_0_, *A*_line_) within fixed absolute differences to the MoCap were calculated as follows: *n*(|Vmax-MoCap| ≤ *x*)/ *n*(|Vmax-MoCap|) · 100, where *n* is defined as the number of measures and *x* is defined as the fixed absolute difference. The following fixed absolute differences were used to provide a range of practically relevant deviations (in m · s^−1^) between Vmax and the MoCap: ≤ 0.01, ≤ 0.02, ≤ 0.05, ≤ 0.1, ≤ 0.2 for the velocities during the 1RM test, the submaximal sessions and *v*_0_. Furthermore, for the predicted *L*_0_ and the 1RM the following fixed absolute differences (in kg) were used: ≤ 1, ≤ 3, ≤ 5, ≤ 7, ≤ 10, while for the Aline the proportion of Vmax data is displayed for the following absolute differences (in m · s^−1^ · kg): ≤ 0.5, ≤ 1, ≤ 2, ≤ 5, ≤ 10.

For the determination of intra-day reliability, we evaluated the MV assessed at each load (i.e., 30, 50, 70 and 90% 1RM) within each session (3 repetitions at each load, separately during sessions 2–4). Additionally, for inter-day reliability, we evaluated the mean MV at each load (i.e., 30, 50, 70 and 90% 1RM) between the submaximal experimental sessions (2–4). For both inter- and intra-day reliability, coefficients of variation (CV) were calculated for each individual. Since the CV of MoCap indicates the actual variance (i.e. biological variance) between the repetitions, the absolute difference of the CVs between MoCap and Vmaxpro represents the variance caused by the Vmaxpro. Therefore, the ‘true’ CVs for the Vmaxpro were calculated as follows: CV_Vmaxpro_ = (|absCV_Vmax-_CV_MoCap_|), where CVs < 10% were considered as a measure for acceptable reliability [17]. All CVs were calculated in Excel (Microsoft Corporation, version 2201, Redmond, USA), while all other statistical analyses were performed using SPSS (IBM SPSS Statistics, version 28, Chicago, IL). Statistical significance for all tests was set a *p* ≤ 0.05.

## RESULTS

### Validity – incremental 1RM test

**FIG. 3:**
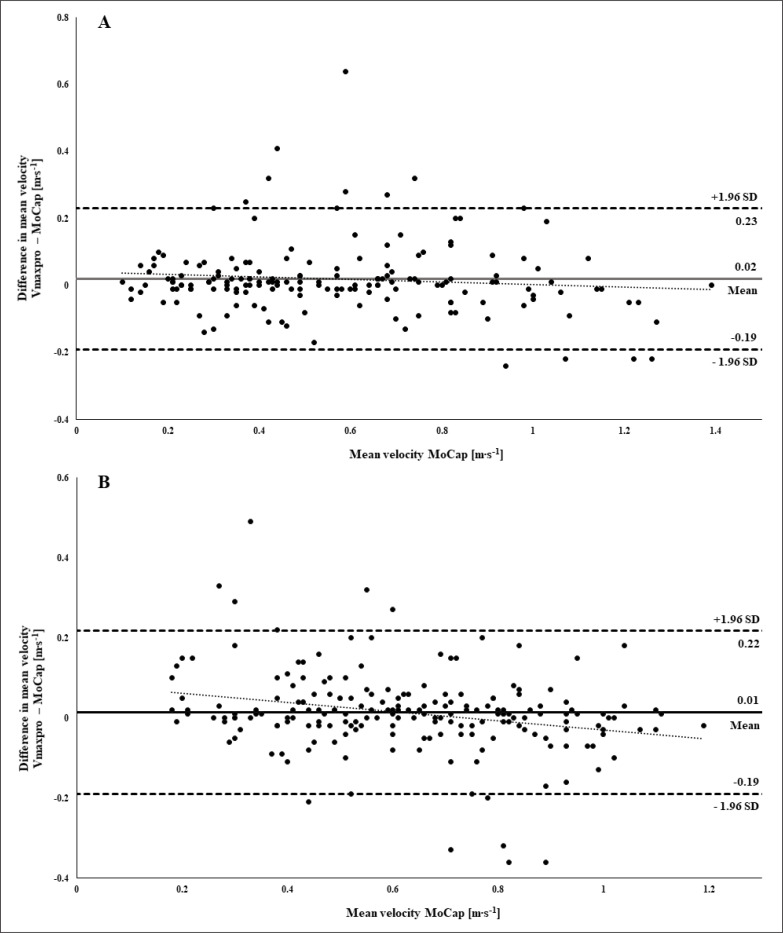
Bland-Altman analysis with limits of agreement for bench press (A) and squat (B) data (± 1.96 SD).

For BP and SQ, 170 out of 197 repetitions (85.9%) and 197 out of 208 repetitions (94.7%), respectively, were assessed by Vmaxpro and hence included in the validity analysis. The distribution of missing data in relation to different MVs is illustrated in Figure S1. The mean bias of the MVs assessed by the Vmaxpro and MoCap was 0.02 m · s^−1^ (standard deviation [SD]: 0.11 m · s^−1^; limits of agreement [LoA]: 0.21 m · s^−1^) and 0.01 m · s^−1^ (SD: 0.11 m · s^−1^; LoA: 0.21 m · s^−1^) for BP and SQ, respectively (Figure 3). The Pearson product-moment correlation coefficient *r* was 0.935 and 0.900 (both *p* ≤ 0.01) for BP and SQ, respectively. Regression analyses revealed a statistically significant linear relation between the Vmaxpro and the MoCap BP (F(1, 171) = 1175.52; *p* ≤ 0.01; *R*^2^ = 0.874) and SQ (F(1, 197) = 835.82; *p* ≤ 0.01; *R*^2^ = 0.810). When comparing the differences of the criterion and index (i.e., Vmaxpro vs. MoCap) with the MV of the criterion, regression analyses showed a statistically significant linear association for BP (F(1, 153) = 11.81; *p* = 0.001; *R*^2^ = 0.072) with f(∆Vmaxpro -MoCap) = -0.0389x + 0.042 and SQ (F(1, 197) = 7.31; *p* = 0.007; *R*^2^ = 0.031) with f(∆Vmaxpro -MoCap) = -0.1142x + 0.085 (Figure 3). The mean MAPE across all loads for Vmaxpro compared with the MoCap was 12.32 ± 15.03% and 11.94 ± 15.91% for BP and SQ, respectively. The proportion of Vmax data obtained from the 1RM test within fixed absolute differences compared to the MoCap is displayed in Table 1.

**TABLE 1:** Proportion of Vmax data [%] within fixed absolute difference compared to the MoCap.

**velocities during 1RM–test [m · s-1]**					
**+/-**	≤ **0.01**	≤ **0.02**	≤ **0.05**	≤ **0.1**	≤ **0.2**
**BP**	26.05	36.74	49.30	65.12	73.49
**SQ**	21.86	37.67	57.67	74.42	86.98

**velocities during submaximal session with 30% 1RM [m · s-1]**					
**+/-**	≤ **0.01**	≤ **0.02**	≤ **0.05**	≤ **0.1**	≤ **0.2**
**BP**	15.75	23.97	48.63	73.29	84.25
**SQ**	19.18	30.82	63.70	84.25	93.84

**velocities during submaximal session with 50% 1RM [m · s-1]**					
**+/-**	≤ **0.01**	≤ **0.02**	≤ **0.05**	≤ **0.1**	≤ **0.2**
**BP**	23.97	39.73	67.12	78.77	89.04
SQ	18.49	32.19	67.12	89.04	96.58

**velocities during submaximal session with 70% 1RM [m · s-1]**					
**+/-**	≤ **0.01**	≤ **0.02**	≤ **0.05**	≤ **0.1**	≤ **0.2**
**BP**	34.46	56.76	82.43	95.27	98.65
**SQ**	28.38	45.95	79.73	91.22	97.97

**velocities during submaximal session with 90% 1RM [m · s-1]**					
**+/-**	≤ **0.01**	≤ **0.02**	≤ **0.05**	≤ **0.1**	≤ **0.2**
**BP**	53.57	68.57	86.43	98.57	100.00
**SQ**	37.86	58.57	83.57	92.14	97.86

***L*_0_ [kg]**					
**+/-**	≤ **1**	≤ **3**	≤ **5**	≤ **7**	≤ **10**
**BP**	22.00	44.00	70.00	86.00	88.00
**SQ**	10.00	22.00	32.00	42.00	54.00

***v*_0_ [m · s^-1^]**					
**+/-**	≤ **0.01**	≤ **0.02**	≤ **0.05**	≤ **0.1**	≤ **0.2**
**BP**	12.00	18.00	40.00	62.00	88.00
**SQ**	10.00	16.00	38.00	72.00	90.00

***A*_line_ [m · s^-1^ · kg]**					
**+/-**	≤ **0.5**	≤ **1**	≤ **2**	≤ **5**	≤ **10**
**BP**	22.00	30.00	64.00	88.00	98.00
**SQ**	10.00	16.00	30.00	54.00	74.00

**1RM [kg]**					
**+/-**	≤ **1**	≤ **3**	≤ **5**	≤ **7**	≤ **10**
**BP**	22.00	52.00	78.00	88.00	90.00
**SQ**	12.00	30.00	48.00	50.00	66.00

### Validity – L-V variables and 1RM

**FIG. 4:**
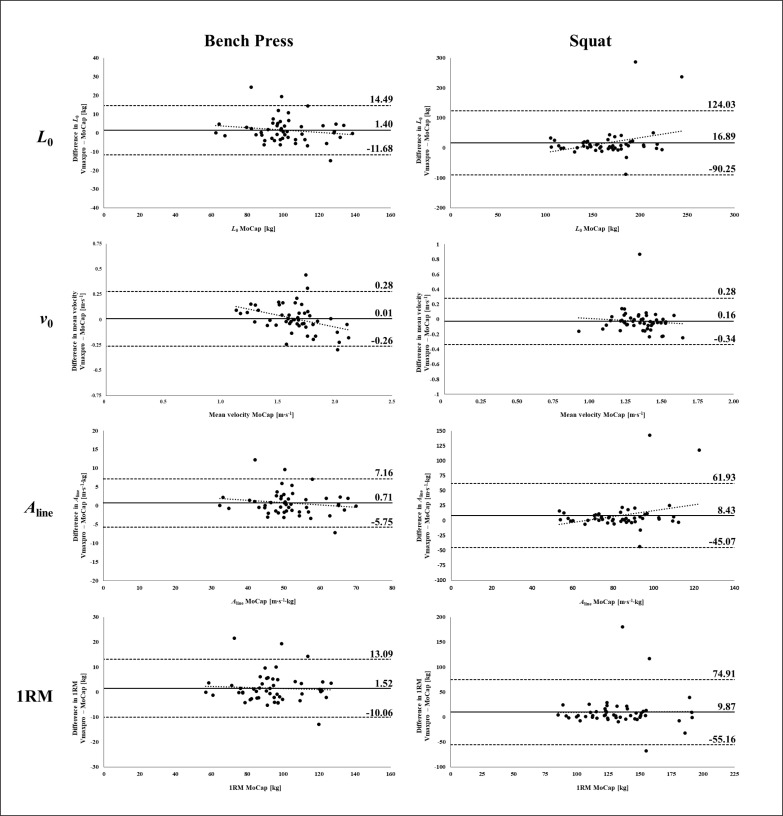
Bland-Altman analysis with limits of agreement (± 1.96 SD) for both bench press and squat *L-V* variables and 1RM.

The mean bias and the LoA for the calculated 1RM and *L-V* variables by the index and criterion are displayed in Figure 4. The Pearson product-moment correlation coefficients *r* for the calculated *L-V* variables and the 1RM between the index and criterion ranged from 0.808 to 0.942 (all *p* ≤ 0.01) and from 0.615 to 0.741 (all *p* ≤ 0.01) for BP and SQ, respectively (Table 2). The *R*^2^ ranged from 0.652 to 0.887 (all *p* ≤ 0.001) and from 0.378 to 0.548 (all *p* ≤ 0.001) for BP and SQ, respectively. The proportion of the calculated *L-V* variables and the 1RM based on the Vmax data within fixed absolute differences compared to the MoCap is displayed in Table 1.

**TABLE 2:** Mean values of the *L-V* variables and the predicted 1RM with the respective r, R^2^ and mean absolute percentage error (MAPE) for Vmax and the MoCap. BP = bench press, SQ = squat.

**BP**	**Vmax**	**MoCap**	***r***	***R* ^2^**	**MAPE [%]**
*L*_0_ [kg]	102.98 ± 17.38	101.58 ± 17.18	0.925 (≤ 0.001^**^)	0.856 (≤ 0.001^**^)	4.46 ± 4.40
*v*_0_ [m · s^−1^]	1.66 ± 0.22	1.65 ± 0.23	0.808 (≤ 0.001^**^)	0.652 (≤ 0.001^**^)	6.01 ± 5.07
*A*_line_ [m · s^−1^ · kg]	52.32 ± 8.66	51.61 ± 8.56	0.927 (≤ 0.001^**^)	0.859 (≤ 0.001^**^)	4.30 ± 4.30
1RM [kg]	95.12 ± 17.58	93.60 ± 16.90	0.942 (≤ 0.001^**^)	0.887 (≤ 0.001^**^)	4.06 ± 4.31

**SQ**	**Vmax**	**MoCap**	***r***	***R* ^2^**	**MAPE [%]**
*L*_0_ [kg]	182.76 ± 70.71	165.87 ± 31.69	0.740 (≤ 0.001^**^)	0.548 (≤ 0.001^**^)	10.65 ± 16.18
*v*_0_ [m · s^−1^]	1.32 ± 0.20	1.35 ± 0.14	0.615 (≤ 0.001^**^)	0.378 (≤ 0.001^**^)	7.00 ± 7.18
*A*_line_ [m · s^−1^ · kg]	92.04 ± 35.30	83.61 ± 15.84	0.741 (≤ 0.001^**^)	0.549 (≤ 0.001^**^)	10.51 ± 15.86
1RM [kg]	142.96 ± 42.59	133.09 ± 26.25	0.627 (≤ 0.001^**^)	0.393 (≤ 0.001^**^)	9.48 ± 14.30

### Reliability – submaximal experimental session

The results of the intra-day and inter-day reliability analysis for both exercises are displayed in Tables 3 and 4, respectively. Mean intra-day CVs ranged from 2.4% to 9.7% and 3.7% to 8.6% for BP and SQ, respectively. Mean inter-day CVs ranged from 3.5% to 5.9% and 3.2% to 6.7% for BP and SQ, respectively.

**TABLE 3:** Mean MV and intra-day coefficient of variance (CV) of repetitions 1–3 in sessions 2–4 separated by the different intensities and exercises. BP = bench press, SQ = squat.

	**Session 2**		**Session 3**		**Session 4**	
**Vmax BP**	**MV [m · s^-1^]**	**CV [%]**	**MV [m · s^-1^]**	**CV [%]**	**MV [m · s^-1^]**	**CV [%]**
30%	1.12 ± 0.16	8.7 ± 9.9	1.10 ± 0.21	7.3 ± 7.4	1.10 ± 0.23	9.7 ± 15.5
50%	0.80 ± 0.13	8.7 ± 8.9	0.84 ± 0.11	6.2 ± 7.2	0.82 ± 0.11	5.6 ± 5.8
70%	0.58 ± 0.11	3.7 ± 4.3	0.57 ± 0.09	5.4 ± 6.9	0.57 ± 0.09	4.3 ± 5.2
90%	0.34 ± 0.14	2.4 ± 1.7	0.32 ± 0.07	4.5 ± 5.8	0.30 ± 0.07	5.6 ± 7.5

**Vmax BP**	**MV [m · s^-1^]**	**CV [%]**	**MV [m · s^-1^]**	**CV [%]**	**MV [m · s^-1^]**	**CV [%]**
30%	0.95 ± 0.18	7.8 ± 13.4	0.95 ± 0.17	8.6 ± 13.8	1.00 ± 0.15	6.3 ± 9.7
50%	0.79 ± 0.14	3.9 ± 4.5	0.78 ± 0.15	4.8 ± 7.1	0.81 ± 0.10	4.6 ± 8.8
70%	0.64 ± 0.07	3.7 ± 3.4	0.64 ± 0.08	3.9 ± 4.8	0.64 ± 0.14	4.7 ± 7.4
90%	0.45 ± 0.09	4.4 ± 5.4	0.45 ± 0.08	4.8 ± 5.9	0.44 ± 0.13	5.5 ± 7.8

**TABLE 4:** Mean MV and inter-day coefficient of variance (CV) between sessions 2–4 separated by the different intensities and exercises. BP = bench press, SQ = squat.

	Mean velocity [m · s–1]			CV [%]
**Vmax BP**	**Session 2**	**Session 3**	**Session 4**	
30%	1.12 ± 0.13	1.10 ± 0.15	1.10 ± 0.17	5.9 ± 7.5
50%	0.80 ± 0.11	0.86 ± 0.11	0.82 ± 0.10	5.6 ± 6.9
70%	0.58 ± 0.10	0.57 ± 0.07	0.56 ± 0.09	3.5 ± 3.1
90%	0.45 ± 0.08	0.44 ± 0.07	0.44 ± 0.12	5.6 ± 9.0

**Vmax SQ**	**Session 2**	**Session 3**	**Session 4**	
30%	0.95 ± 0.12	0.95 ± 0.14	0.99 ± 0.11	3.7 ± 5.7
50%	0.81 ± 0.06	0.79 ± 0.08	0.81 ± 0.08	3.2 ± 3.0
70%	0.64 ± 0.06	0.64 ± 0.07	0.66 ± 0.10	3.2 ± 4.6
90%	0.45 ± 0.08	0.44 ± 0.07	0.44 ± 0.12	6.7 ± 10.8

## DISCUSSION

The aim of the study was to assess the ecological validity, as well as intra-day and inter-day reliability, of the Vmaxpro sensor during a 1RM test and at submaximal loads (i.e., 30, 50, 70 and 90% of the 1RM) using free weights in both BP and SQ. Additionally, to gain a better understanding of how the possible deviation of the Vmaxpro influences training practice, we examined the validity of the Vmaxpro to predict *L-V* relationship variables (i.e., *L*_0_, *v*_0_, *A*_line_) and the 1RM in both exercises. The validity analysis revealed a nearly perfect correlation between data derived from the Vmaxpro and MoCap for both exercises. However, compared to the MoCap, the Vmaxpro showed a systematic overestimation of the MV across all loads that is decreasing with higher MVs in BP and SQ. The comparison between the *L-V* variables and the 1RM derived from Vmaxpro and the MoCap showed a very high to nearly perfect and a high to very high validity for BP and SQ with a systematic overestimation for all variables. The Bland-Altman analysis, however, indicated high LoA, particularly for the SQ *L-V* variables and the 1RM. The CVs for the intra-day and inter-day reliability of the Vmaxpro were within an acceptable range for all loads in both exercises.

Compared with our previous data on the Vmaxpro to assess the MV validity during a guided barbell SQ [10], our present data indicate lower validity when using free weights (*R*^2^ = 0.935 vs. 0.810). This, however, can at least partially be explained by the degrees of freedom (3 axes vs. 1 axis). It appears that the Vmaxpro is not able to detect the changes in movement trajectory during free weight exercises with sufficient accuracy. In contrast to our findings, a recent study examining the validity of the Vmaxpro for the assessment of the MV during a free-weight SQ and hip thrusts reported good to excellent validity, indicated by low LoA (0.1 m · s^−1^ and 0.12 m · s^−1^ for SQ and hip thrusts, respectively) [18]. However, it needs to be considered that this study used a linear position transducer but not the gold standard (optical 3D motion capture system) as the criterion. Therefore, these results should be interpreted with caution. When comparing the validity of the Vmaxpro to assess the MV in both exercises, the LoA during the 1RM test in our study show comparable validity for BP and SQ (0.21 m · s^−1^ vs. 0.20 m · s^−1^). To the best of our knowledge, there are no existing data on the validity of the Vmax-pro to assess the MV in free weight BP exercise. However, when compared to another IMU (i.e., PUSH Band), the Vmaxpro showed a lower mean bias, but higher LoA (mean bias: 0.10 ± 0.06 m · s^−1^, LoA: 0.13 (extracted with the WebPlotDigitizer, Pacifica, California, USA, Version: 4.4) [19]) for assessing MV during a 1RM test [20]. When compared to data on the validity to the Beast sensor [20, 21], the Vmaxpro demonstrated higher validity in both BP and SQ. Regardless of the exercise, the validity of the Vmaxpro is comparable or higher, when compared to other commercially available IMUs [9].

In line with a previous study [10], we found a slight overestimation of the MV compared with the MoCap for both BP and SQ during the 1RM test. However, contrarily to our previous results, the systematic bias decreased with higher MVs, indicating higher validity at higher MVs (i.e., lower loads). A potential explanation for poorer validity at lower MVs remains speculative at this point but could be related to a larger variance in the movement trajectory during slower repetitions. These conflicting results do, however, rein-force the arbitrariness regarding the systematic over/underestimation at different MVs within the same IMU that we described previously [10]. Furthermore, it was intriguing that a high proportion of repetitions (14.1% and 5.3% for BP and SQ, respectively) during the 1RM test was not assessed by the Vmaxpro. As indicated by Figure S1, this appeared to be mostly the case at low MVs during BP. Obviously, a justification for this cannot be given but it needs to be noted that the manufacturer’s user manual specifies that only MVs > 0.15 m · s^−1^ are detected by the sensor. In our data, however, this was only the case for 9 of the 36 non-acquired data points. This high proportion of missing data, especially for BP, is a major limitation of the sensor and should be considered when using the sensor in practice.

In order to provide valuable information on how the possible deviation of the Vmaxpro influences training practice, the proportion of Vmax data within fixed absolute differences to the MoCap was calculated. For example, during velocity-based training (VBT) with the fastest repetition at 0.5 m · s^−1^ and a typically used velocity threshold of 20% [22], an underestimation of the MV of 0.1 m · s^−1^ could already lead to a termination of the set, even without an actual loss in MV. In our study, MVs around 0.5 m · s^−1^ were reached at loads of 70% and 90% of the 1RM. The majority of MVs (~ 80%) assessed by the Vmaxpro at these loads are, however, within an acceptable absolute difference (≤ 0.05 m · s^−1^) to the MoCap. Thus, it seems that the Vmaxpro could be a valid IMU for assessing the MVs during VBT for recreational purposes. However, whether the accuracy of the sensor is sufficient for individual requirements has to be judged in a case-specific manner. Especially when using VBT for the development of explosive strength determinants, an exact estimation of the MV is necessary to avoid undesired fatigue caused by an over-estimation of the MV. Therefore, practitioners should consider the absolute deviation in MV between the Vmaxpro and the gold standard (displayed in Table 1) to estimate whether this deviation could negatively influence the chronic development of the desired strength training parameter and based on that to evaluate whether use of the Vmaxpro is helpful in training practice.

For BP and SQ, the correlation between the *L-V* variables and the 1RM derived from Vmaxpro and the MoCap was very high to nearly perfect and high to very high, respectively. However, the Bland-Altman analysis indicated high LoA for the SQ *L-V* variables and the 1RM (e.g. 55.16 to 74.91 kg for the 1RM) with a systematic over-estimation (e.g. 9.87 kg for the 1RM). This overestimation can be explained, to some extent, by a limited number of extreme outliers in the Bland-Altman plots (Figure 4). In turn, it needs to be considered that the biological inter-day variance of the MV for submaximal loads [23] affects the predicted *L-V* variables and the 1RM. This high inter-day variance may exceed the displayed differences between the data derived by the Vmaxpro and the MoCap. Thus, a high proportion of predicted *L-V* variables and the 1RM, especially for the BP, appears to be within an acceptable absolute difference for monitoring these variables. However, practitioners and athletes should be aware of the deviation in both *L-V* variables and the 1RM derived from the Vmaxpro, because in some elite sports a more precise estimation of the 1RM is essential. Furthermore, it needs to be addressed that the *L-V* variables and the 1RM were calculated based on the highest MV of 3 repetitions at each intensity, reducing the influence of possible outliers. Therefore, when aiming to calculate *L-V* variables and the 1RM based on the MVs assessed by the Vmaxpro, practitioners are advised to use more than one repetition for each load.

Regarding the reliability of the Vmaxpro, our study revealed acceptable (< 10%) intra-day and inter-day CVs for all loadings. Therefore, the reliability of the Vmaxpro can be classified as higher compared to the Beast sensor and comparable to the PUSH band [20]. However, the high standard deviation (up to 15.47%), especially for low load intra-day CVs, indicates large variations of the reliability between different measurements/individuals. Whether this can be explained by interindividual differences in the execution of the exercises needs to be addressed in future studies.

When interpreting our data, some limitations need to be considered. Firstly, we used only one Vmaxpro device; therefore, it cannot be ruled out that the observed error was device-specific. Furthermore, we did not use two sensors at the same time; thus, we did not assess the intra-device agreement (i.e. Vmaxpro1 vs. Vmaxpro2). Furthermore, factors such as the strength training experience, anthropometric data and, thus, the range of motion could influence the validity. Future studies should address possible differences in validity, for example in a heterogenous sample including different strength levels and anthropometrics.

## CONCLUSIONS

Taking our findings together, the Vmaxpro seems to have acceptable validity for most recreational purposes. However, the lower validity at higher loads (i.e., lower velocities) may be of concern when using nearly maximal loads and/or using low velocity loss thresholds during VBT. Moreover, it was intriguing that a relatively high number of repetitions during the 1RM test (i.e., 14.1% and 5.3% for bench press and squat, respectively) were not assessed by the Vmaxpro in the present study. Whether this is a common observation specific to the device needs to be assessed in future studies. Also, the wide limits of agreement for the 1RM prediction may be sufficient for recreational purposes but not for elite sport settings where already small deviations may lead to undesired training results. In terms of the reliability, our data indicate the sensor to be suitable for monitoring changes in performance within the same individual in different settings of VBT (i.e., using velocity loss thresholds for training monitoring or assessing chronic changes in movement velocity).

## Supplementary Material

Evaluation of the Vmaxpro sensor for assessing movement velocity and load-velocity variables: accuracy and implications for practical use
