# Supplementary material for: Evaluation of the Vmaxpro sensor for assessing movement velocity and load-velocity variables: accuracy and implications for practical use
Source: Biol Sport. 2023 May 25;41(1):41–51. doi: 10.5114/biolsport.2024.125596 (PMC10765425; doi:10.5114/biolsport.2024.125596)

Supplemental figure

FIG. S1. Distribution of missing SQ and BP data in relation to different MVs, indicating a high proportion of missing data at low MVs, particularly in the BP exercise.

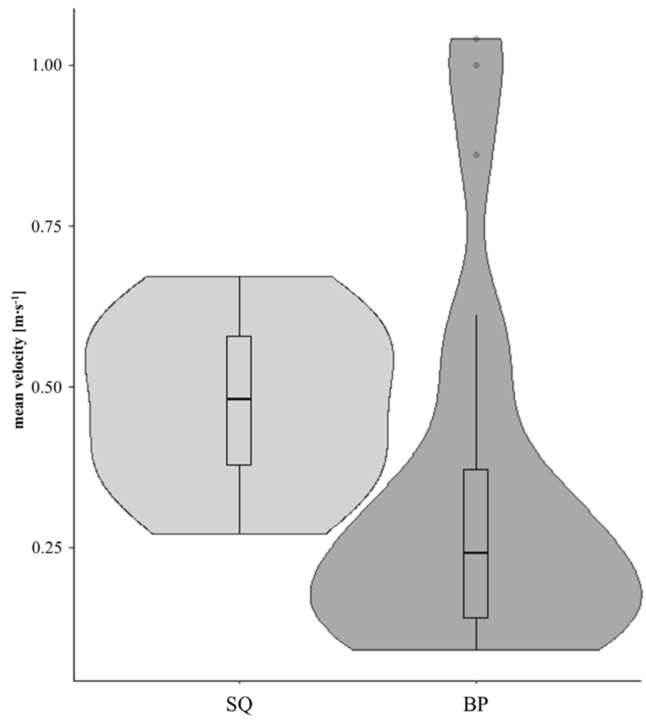

Supplement: Evaluation of the Vmaxpro sensor for assessing movement velocity and load-velocity variables: accuracy and implications for practical use [file JBS-41-50259-s1.pdf]
